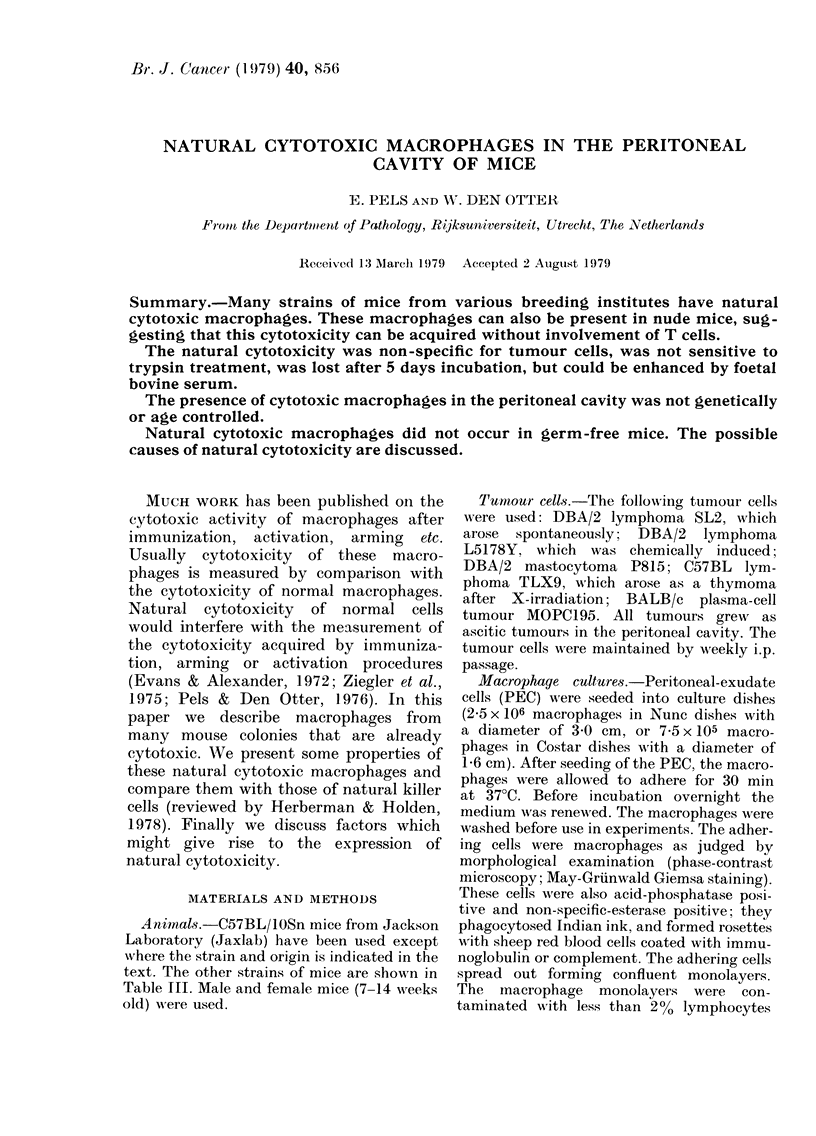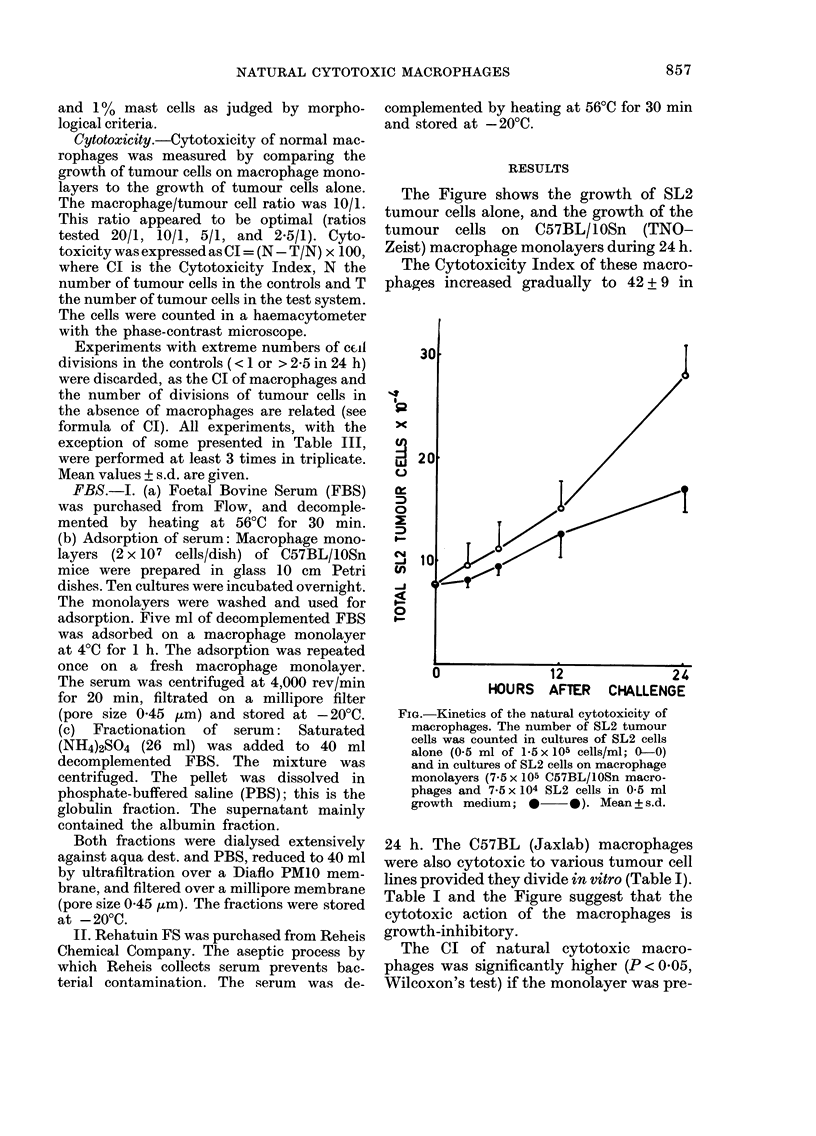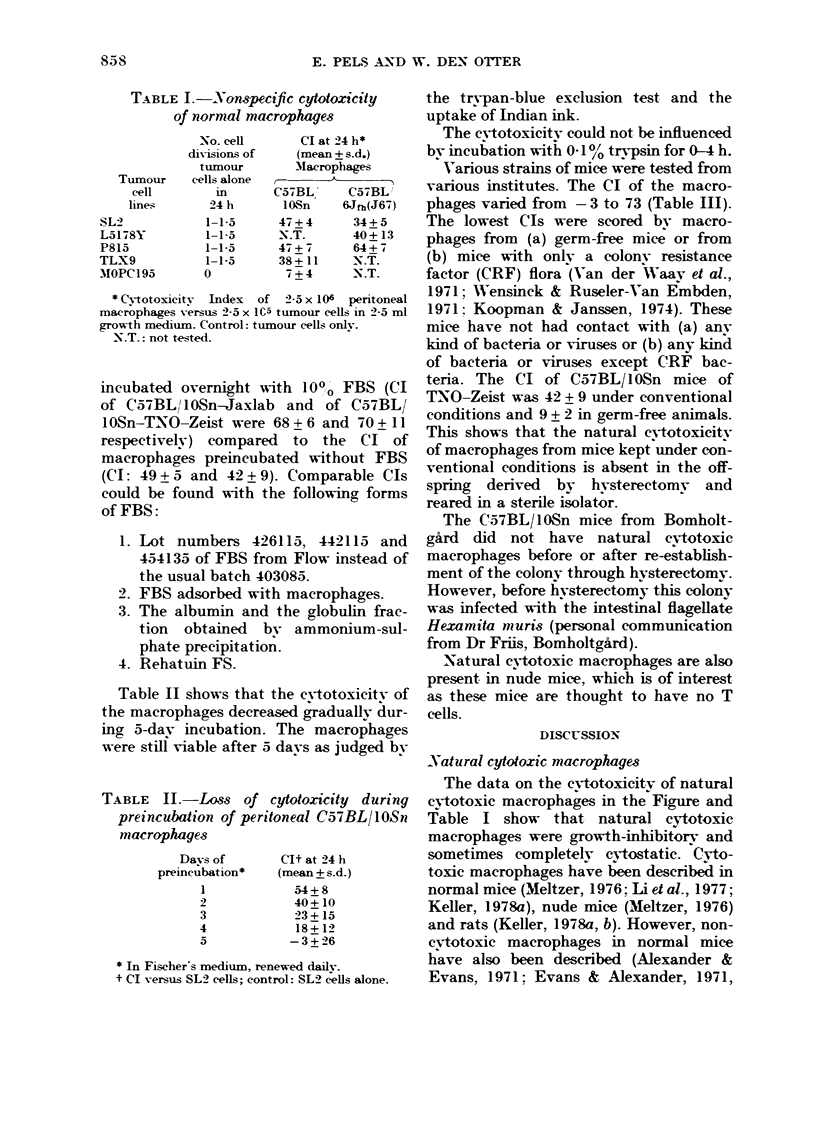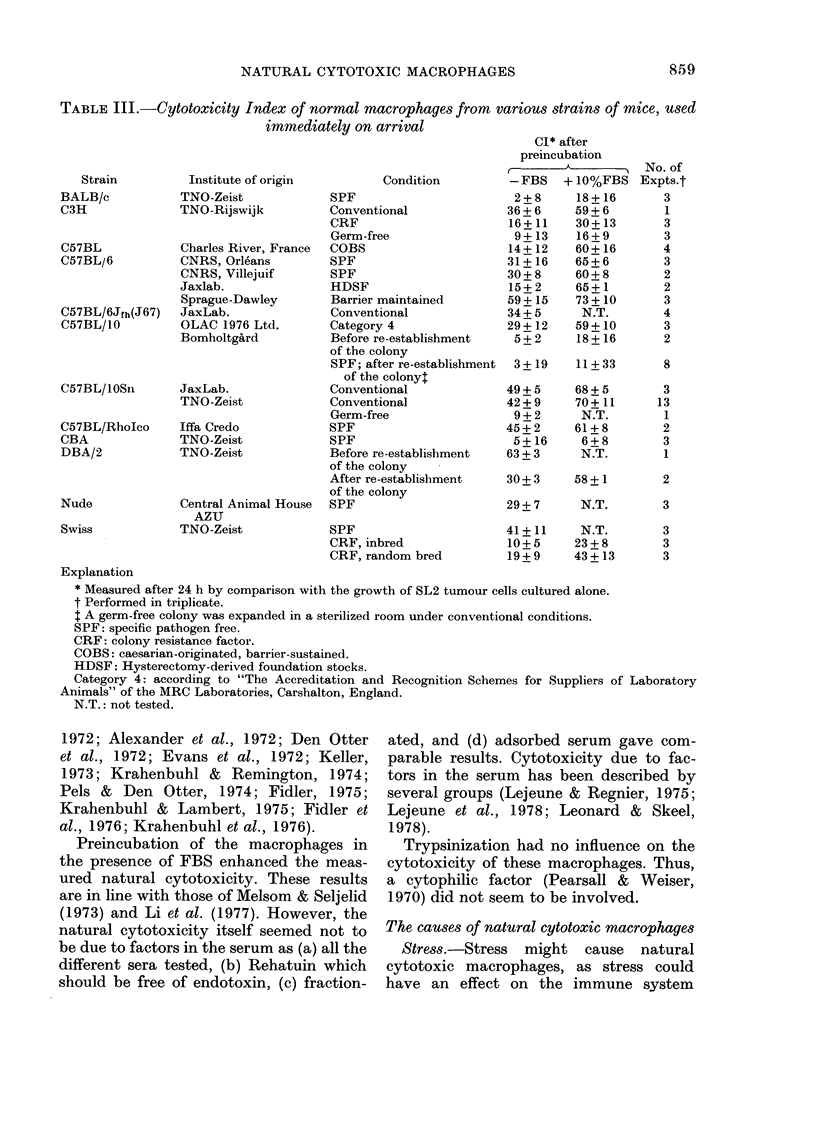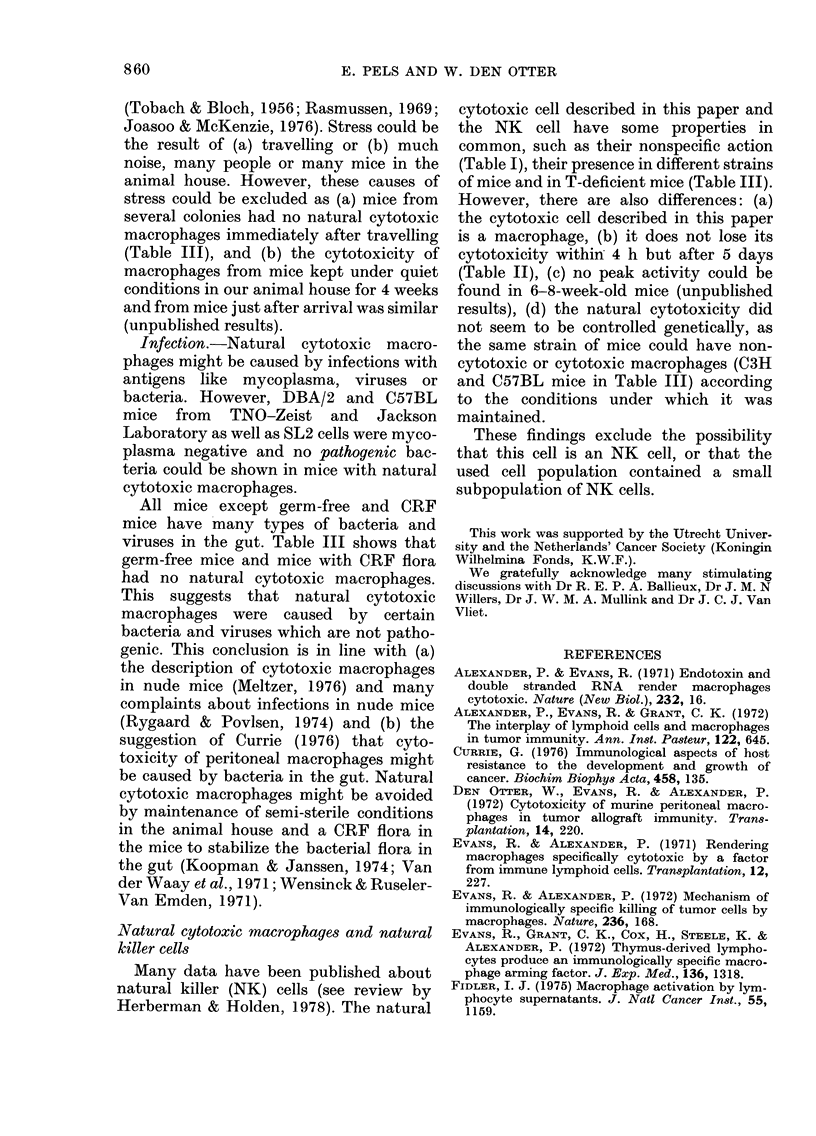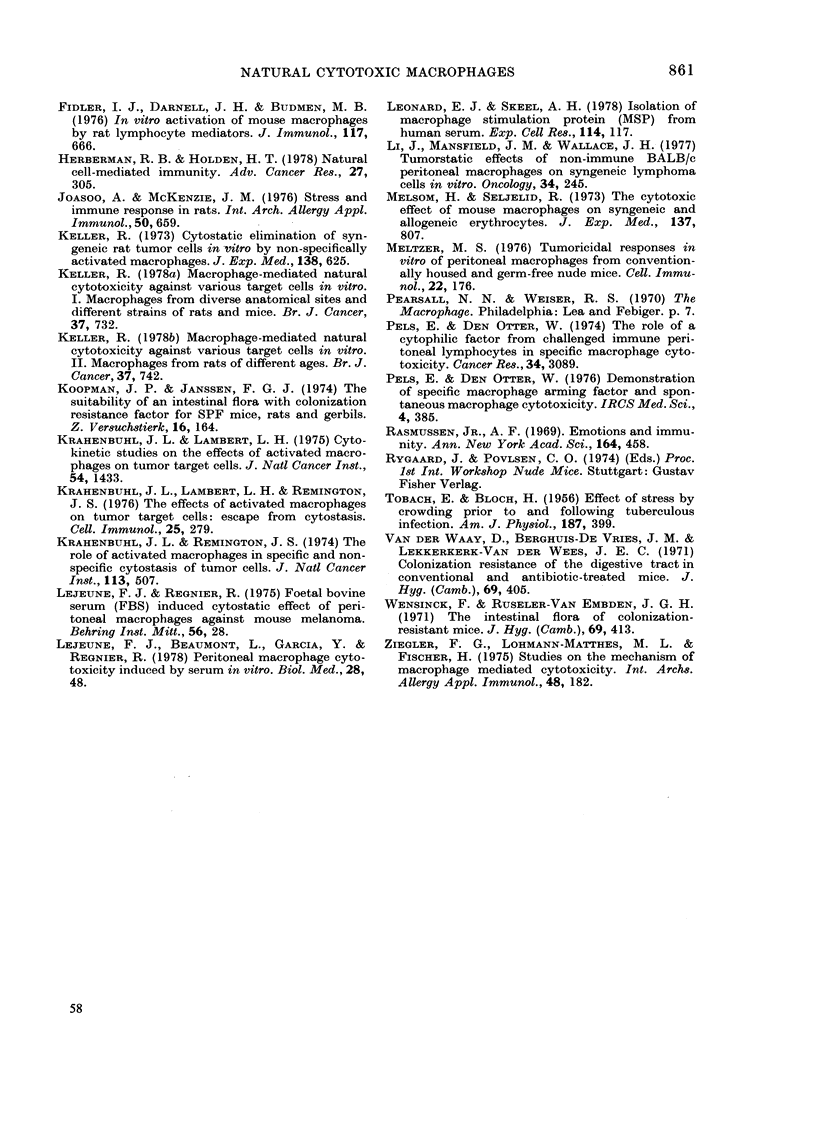# Natural cytotoxic macrophages in the peritoneal cavity of mice.

**DOI:** 10.1038/bjc.1979.276

**Published:** 1979-12

**Authors:** E. Pels, W. Den Otter

## Abstract

Many strains of mice from various breeding institutes have natural cytotoxic macrophages. These macrophages can also be present in nude mice, suggesting that this cytotoxicity can be acquired without invovlvement of T cells. The natural cytotoxicity was non-specific for tumour cells, was not sensitive to trypsin treatment, was lost after 5 days incubation, but could be enhanced by foetal bovine serum. The presence of cytotoxic macrophages in the peritoneal cavity was not genetically or age controlled. Natural cytotoxic macrophages did not occur in germ-free mice. The possible causes of natural cytotoxicity are discussed.


*Br. J. Cancer* (1979) **40**, 856

### NATURAL CYTOTOXIC MACROPHAGES IN THE PERITONEAL CAVITY OF MICE

E. PELS AND W. DEN OTTER

*From the Department of Pathology, Rijksuniversiteit, Utrecht, The Netherlands*

Received 13 March 1979 Accepted 2 August 1979

**Summary.—Many strains of mice from various breeding institutes have natural cytotoxic macrophages. These macrophages can also be present in nude mice, suggesting that this cytotoxicity can be acquired without involvement of T cells.**

**The natural cytotoxicity was non-specific for tumour cells, was not sensitive to trypsin treatment, was lost after 5 days incubation, but could be enhanced by foetal bovine serum.**

**The presence of cytotoxic macrophages in the peritoneal cavity was not genetically or age controlled.**

**Natural cytotoxic macrophages did not occur in germ-free mice. The possible causes of natural cytotoxicity are discussed.**

MUCH WORK has been published on the cytotoxic activity of macrophages after immunization, activation, arming *etc.* Usually cytotoxicity of these macrophages is measured by comparison with the cytotoxicity of normal macrophages. Natural cytotoxicity of normal cells would interfere with the measurement of the cytotoxicity acquired by immunization, arming or activation procedures (Evans & Alexander, 1972; Ziegler *et al.*, 1975; Pels & Den Otter, 1976). In this paper we describe macrophages from many mouse colonies that are already cytotoxic. We present some properties of these natural cytotoxic macrophages and compare them with those of natural killer cells (reviewed by Herberman & Holden, 1978). Finally we discuss factors which might give rise to the expression of natural cytotoxicity.

### MATERIALS AND METHODS

*Animals.*—C57BL/10Sn mice from Jackson Laboratory (Jaxlab) have been used except where the strain and origin is indicated in the text. The other strains of mice are shown in Table III. Male and female mice (7–14 weeks old) were used.

*Tumour cells.*—The following tumour cells were used: DBA/2 lymphoma SL2, which arose spontaneously; DBA/2 lymphoma L5178Y, which was chemically induced; DBA/2 mastocytoma P815; C57BL lymphoma TLX9, which arose as a thymoma after X-irradiation; BALB/c plasma-cell tumour MOPC195. All tumours grew as ascitic tumours in the peritoneal cavity. The tumour cells were maintained by weekly i.p. passage.

*Macrophage cultures.*—Peritoneal-exudate cells (PEC) were seeded into culture dishes ($2{\cdot}5 \times 10^6$ macrophages in Nunc dishes with a diameter of 3·0 cm, or $7{\cdot}5 \times 10^5$ macrophages in Costar dishes with a diameter of 1·6 cm). After seeding of the PEC, the macrophages were allowed to adhere for 30 min at 37°C. Before incubation overnight the medium was renewed. The macrophages were washed before use in experiments. The adhering cells were macrophages as judged by morphological examination (phase-contrast microscopy; May-Grünwald Giemsa staining). These cells were also acid-phosphatase positive and non-specific-esterase positive; they phagocytosed Indian ink, and formed rosettes with sheep red blood cells coated with immunoglobulin or complement. The adhering cells spread out forming confluent monolayers. The macrophage monolayers were contaminated with less than 2% lymphocytes and 1% mast cells as judged by morphological criteria.

*Cytotoxicity*.—Cytotoxicity of normal macrophages was measured by comparing the growth of tumour cells on macrophage monolayers to the growth of tumour cells alone. The macrophage/tumour cell ratio was 10/1. This ratio appeared to be optimal (ratios tested 20/1, 10/1, 5/1, and 2·5/1). Cytotoxicity was expressed as $CI = (N - T/N) \times 100$, where CI is the Cytotoxicity Index, N the number of tumour cells in the controls and T the number of tumour cells in the test system. The cells were counted in a haemacytometer with the phase-contrast microscope.

Experiments with extreme numbers of cell divisions in the controls ($<1$ or $>2{\cdot}5$ in 24 h) were discarded, as the CI of macrophages and the number of divisions of tumour cells in the absence of macrophages are related (see formula of CI). All experiments, with the exception of some presented in Table III, were performed at least 3 times in triplicate. Mean values ± s.d. are given.

*FBS*.—I. (a) Foetal Bovine Serum (FBS) was purchased from Flow, and decomplemented by heating at 56°C for 30 min. (b) Adsorption of serum: Macrophage monolayers ($2 \times 10^7$ cells/dish) of C57BL/10Sn mice were prepared in glass 10 cm Petri dishes. Ten cultures were incubated overnight. The monolayers were washed and used for adsorption. Five ml of decomplemented FBS was adsorbed on a macrophage monolayer at 4°C for 1 h. The adsorption was repeated once on a fresh macrophage monolayer. The serum was centrifuged at 4,000 rev/min for 20 min, filtrated on a millipore filter (pore size 0·45 $\mu$m) and stored at −20°C. (c) Fractionation of serum: Saturated $(NH_4)_2SO_4$ (26 ml) was added to 40 ml decomplemented FBS. The mixture was centrifuged. The pellet was dissolved in phosphate-buffered saline (PBS); this is the globulin fraction. The supernatant mainly contained the albumin fraction.

Both fractions were dialysed extensively against aqua dest. and PBS, reduced to 40 ml by ultrafiltration over a Diaflo PM10 membrane, and filtered over a millipore membrane (pore size 0·45 $\mu$m). The fractions were stored at −20°C.

II. Rehatuin FS was purchased from Reheis Chemical Company. The aseptic process by which Reheis collects serum prevents bacterial contamination. The serum was decomplemented by heating at 56°C for 30 min and stored at −20°C.

### RESULTS

The Figure shows the growth of SL2 tumour cells alone, and the growth of the tumour cells on C57BL/10Sn (TNO-Zeist) macrophage monolayers during 24 h.

The Cytotoxicity Index of these macrophages increased gradually to 42 ± 9 in

FIG.—Kinetics of the natural cytotoxicity of macrophages. The number of SL2 tumour cells was counted in cultures of SL2 cells alone (0·5 ml of $1{\cdot}5 \times 10^5$ cells/ml; O—O) and in cultures of SL2 cells on macrophage monolayers ($7{\cdot}5 \times 10^5$ C57BL/10Sn macrophages and $7{\cdot}5 \times 10^4$ SL2 cells in 0·5 ml growth medium; ●——●). Mean ± s.d.

24 h. The C57BL (Jaxlab) macrophages were also cytotoxic to various tumour cell lines provided they divide *in vitro* (Table I). Table I and the Figure suggest that the cytotoxic action of the macrophages is growth-inhibitory.

The CI of natural cytotoxic macrophages was significantly higher ($P < 0{\cdot}05$, Wilcoxon's test) if the monolayer was pre-

TABLE I.—*Nonspecific cytotoxicity of normal macrophages*

| Tumour cell lines | No. cell divisions of tumour cells alone in 24 h | CI at 24 h* (mean ± s.d.) Macrophages C57BL/10Sn | C57BL/6J$_{fh}$(J67) |
|---|---|---|---|
| SL2 | 1-1·5 | 47 ± 4 | 34 ± 5 |
| L5178Y | 1-1·5 | N.T. | 40 ± 13 |
| P815 | 1-1·5 | 47 ± 7 | 64 ± 7 |
| TLX9 | 1-1·5 | 38 ± 11 | N.T. |
| MOPC195 | 0 | 7 ± 4 | N.T. |

* Cytotoxicity Index of 2·5 × 10⁶ peritoneal macrophages versus 2·5 × 10⁵ tumour cells in 2·5 ml growth medium. Control: tumour cells only.

N.T.: not tested.

incubated overnight with 10% FBS (CI of C57BL/10Sn–Jaxlab and of C57BL/10Sn–TNO–Zeist were 68 ± 6 and 70 ± 11 respectively) compared to the CI of macrophages preincubated without FBS (CI: 49 ± 5 and 42 ± 9). Comparable CIs could be found with the following forms of FBS:

1. Lot numbers 426115, 442115 and 454135 of FBS from Flow instead of the usual batch 403085.
2. FBS adsorbed with macrophages.
3. The albumin and the globulin fraction obtained by ammonium-sulphate precipitation.
4. Rehatuin FS.

Table II shows that the cytotoxicity of the macrophages decreased gradually during 5-day incubation. The macrophages were still viable after 5 days as judged by the trypan-blue exclusion test and the uptake of Indian ink.

TABLE II.—*Loss of cytotoxicity during preincubation of peritoneal C57BL/10Sn macrophages*

| Days of preincubation* | CI† at 24 h (mean ± s.d.) |
|---|---|
| 1 | 54 ± 8 |
| 2 | 40 ± 10 |
| 3 | 23 ± 15 |
| 4 | 18 ± 12 |
| 5 | − 3 ± 26 |

* In Fischer's medium, renewed daily.

† CI versus SL2 cells; control: SL2 cells alone.

The cytotoxicity could not be influenced by incubation with 0·1% trypsin for 0–4 h.

Various strains of mice were tested from various institutes. The CI of the macrophages varied from − 3 to 73 (Table III). The lowest CIs were scored by macrophages from (a) germ-free mice or from (b) mice with only a colony resistance factor (CRF) flora (Van der Waay *et al.*, 1971; Wensinck & Ruseler-Van Embden, 1971; Koopman & Janssen, 1974). These mice have not had contact with (a) any kind of bacteria or viruses or (b) any kind of bacteria or viruses except CRF bacteria. The CI of C57BL/10Sn mice of TNO–Zeist was 42 ± 9 under conventional conditions and 9 ± 2 in germ-free animals. This shows that the natural cytotoxicity of macrophages from mice kept under conventional conditions is absent in the offspring derived by hysterectomy and reared in a sterile isolator.

The C57BL/10Sn mice from Bomholtgård did not have natural cytotoxic macrophages before or after re-establishment of the colony through hysterectomy. However, before hysterectomy this colony was infected with the intestinal flagellate *Hexamita muris* (personal communication from Dr Friis, Bomholtgård).

Natural cytotoxic macrophages are also present in nude mice, which is of interest as these mice are thought to have no T cells.

### DISCUSSION

### *Natural cytotoxic macrophages*

The data on the cytotoxicity of natural cytotoxic macrophages in the Figure and Table I show that natural cytotoxic macrophages were growth-inhibitory and sometimes completely cytostatic. Cytotoxic macrophages have been described in normal mice (Meltzer, 1976; Li *et al.*, 1977; Keller, 1978*a*), nude mice (Meltzer, 1976) and rats (Keller, 1978*a*, *b*). However, noncytotoxic macrophages in normal mice have also been described (Alexander & Evans, 1971; Evans & Alexander, 1971,

TABLE III.—*Cytotoxicity Index of normal macrophages from various strains of mice, used immediately on arrival*

| Strain | Institute of origin | Condition | CI* after preincubation −FBS | CI* after preincubation +10%FBS | No. of Expts.† |
|---|---|---|---|---|---|
| BALB/c | TNO-Zeist | SPF | 2±8 | 18±16 | 3 |
| C3H | TNO-Rijswijk | Conventional | 36±6 | 59±6 | 1 |
| | | CRF | 16±11 | 30±13 | 3 |
| | | Germ-free | 9±13 | 16±9 | 3 |
| C57BL | Charles River, France | COBS | 14±12 | 60±16 | 4 |
| C57BL/6 | CNRS, Orléans | SPF | 31±16 | 65±6 | 3 |
| | CNRS, Villejuif | SPF | 30±8 | 60±8 | 2 |
| | Jaxlab. | HDSF | 15±2 | 65±1 | 2 |
| | Sprague-Dawley | Barrier maintained | 59±15 | 73±10 | 3 |
| C57BL/6J$_{fh}$(J67) | JaxLab. | Conventional | 34±5 | N.T. | 4 |
| C57BL/10 | OLAC 1976 Ltd. | Category 4 | 29±12 | 59±10 | 3 |
| | Bomholtgård | Before re-establishment of the colony | 5±2 | 18±16 | 2 |
| | | SPF; after re-establishment of the colony‡ | 3±19 | 11±33 | 8 |
| C57BL/10Sn | JaxLab. | Conventional | 49±5 | 68±5 | 3 |
| | TNO-Zeist | Conventional | 42±9 | 70±11 | 13 |
| | | Germ-free | 9±2 | N.T. | 1 |
| C57BL/RhoIco | Iffa Credo | SPF | 45±2 | 61±8 | 2 |
| CBA | TNO-Zeist | SPF | 5±16 | 6±8 | 3 |
| DBA/2 | TNO-Zeist | Before re-establishment of the colony | 63±3 | N.T. | 1 |
| | | After re-establishment of the colony | 30±3 | 58±1 | 2 |
| Nude | Central Animal House AZU | SPF | 29±7 | N.T. | 3 |
| Swiss | TNO-Zeist | SPF | 41±11 | N.T. | 3 |
| | | CRF, inbred | 10±5 | 23±8 | 3 |
| | | CRF, random bred | 19±9 | 43±13 | 3 |

Explanation

* Measured after 24 h by comparison with the growth of SL2 tumour cells cultured alone.
† Performed in triplicate.
‡ A germ-free colony was expanded in a sterilized room under conventional conditions.
SPF: specific pathogen free.
CRF: colony resistance factor.
COBS: caesarian-originated, barrier-sustained.
HDSF: Hysterectomy-derived foundation stocks.
Category 4: according to "The Accreditation and Recognition Schemes for Suppliers of Laboratory Animals" of the MRC Laboratories, Carshalton, England.
N.T.: not tested.

1972; Alexander *et al.*, 1972; Den Otter *et al.*, 1972; Evans *et al.*, 1972; Keller, 1973; Krahenbuhl & Remington, 1974; Pels & Den Otter, 1974; Fidler, 1975; Krahenbuhl & Lambert, 1975; Fidler *et al.*, 1976; Krahenbuhl *et al.*, 1976).

Preincubation of the macrophages in the presence of FBS enhanced the measured natural cytotoxicity. These results are in line with those of Melsom & Seljelid (1973) and Li *et al.* (1977). However, the natural cytotoxicity itself seemed not to be due to factors in the serum as (a) all the different sera tested, (b) Rehatuin which should be free of endotoxin, (c) fractionated, and (d) adsorbed serum gave comparable results. Cytotoxicity due to factors in the serum has been described by several groups (Lejeune & Regnier, 1975; Lejeune *et al.*, 1978; Leonard & Skeel, 1978).

Trypsinization had no influence on the cytotoxicity of these macrophages. Thus, a cytophilic factor (Pearsall & Weiser, 1970) did not seem to be involved.

*The causes of natural cytotoxic macrophages*

*Stress.*—Stress might cause natural cytotoxic macrophages, as stress could have an effect on the immune system

(Tobach & Bloch, 1956; Rasmussen, 1969; Joasoo & McKenzie, 1976). Stress could be the result of (a) travelling or (b) much noise, many people or many mice in the animal house. However, these causes of stress could be excluded as (a) mice from several colonies had no natural cytotoxic macrophages immediately after travelling (Table III), and (b) the cytotoxicity of macrophages from mice kept under quiet conditions in our animal house for 4 weeks and from mice just after arrival was similar (unpublished results).

*Infection.*—Natural cytotoxic macrophages might be caused by infections with antigens like mycoplasma, viruses or bacteria. However, DBA/2 and C57BL mice from TNO–Zeist and Jackson Laboratory as well as SL2 cells were mycoplasma negative and no *pathogenic* bacteria could be shown in mice with natural cytotoxic macrophages.

All mice except germ-free and CRF mice have many types of bacteria and viruses in the gut. Table III shows that germ-free mice and mice with CRF flora had no natural cytotoxic macrophages. This suggests that natural cytotoxic macrophages were caused by certain bacteria and viruses which are not pathogenic. This conclusion is in line with (a) the description of cytotoxic macrophages in nude mice (Meltzer, 1976) and many complaints about infections in nude mice (Rygaard & Povlsen, 1974) and (b) the suggestion of Currie (1976) that cytotoxicity of peritoneal macrophages might be caused by bacteria in the gut. Natural cytotoxic macrophages might be avoided by maintenance of semi-sterile conditions in the animal house and a CRF flora in the mice to stabilize the bacterial flora in the gut (Koopman & Janssen, 1974; Van der Waay *et al.*, 1971; Wensinck & Ruseler-Van Emden, 1971).

### *Natural cytotoxic macrophages and natural killer cells*

Many data have been published about natural killer (NK) cells (see review by Herberman & Holden, 1978). The natural cytotoxic cell described in this paper and the NK cell have some properties in common, such as their nonspecific action (Table I), their presence in different strains of mice and in T-deficient mice (Table III). However, there are also differences: (a) the cytotoxic cell described in this paper is a macrophage, (b) it does not lose its cytotoxicity within 4 h but after 5 days (Table II), (c) no peak activity could be found in 6–8-week-old mice (unpublished results), (d) the natural cytotoxicity did not seem to be controlled genetically, as the same strain of mice could have non-cytotoxic or cytotoxic macrophages (C3H and C57BL mice in Table III) according to the conditions under which it was maintained.

These findings exclude the possibility that this cell is an NK cell, or that the used cell population contained a small subpopulation of NK cells.

This work was supported by the Utrecht University and the Netherlands' Cancer Society (Koningin Wilhelmina Fonds, K.W.F.).

We gratefully acknowledge many stimulating discussions with Dr R. E. P. A. Ballieux, Dr J. M. N Willers, Dr J. W. M. A. Mullink and Dr J. C. J. Van Vliet.

### REFERENCES

Alexander, P. & Evans, R. (1971) Endotoxin and double stranded RNA render macrophages cytotoxic. *Nature (New Biol.)*, **232**, 16.

Alexander, P., Evans, R. & Grant, C. K. (1972) The interplay of lymphoid cells and macrophages in tumor immunity. *Ann. Inst. Pasteur*, **122**, 645.

Currie, G. (1976) Immunological aspects of host resistance to the development and growth of cancer. *Biochim Biophys Acta*, **458**, 135.

Den Otter, W., Evans, R. & Alexander, P. (1972) Cytotoxicity of murine peritoneal macrophages in tumor allograft immunity. *Transplantation*, **14**, 220.

Evans, R. & Alexander, P. (1971) Rendering macrophages specifically cytotoxic by a factor from immune lymphoid cells. *Transplantation*, **12**, 227.

Evans, R. & Alexander, P. (1972) Mechanism of immunologically specific killing of tumor cells by macrophages. *Nature*, **236**, 168.

Evans, R., Grant, C. K., Cox, H., Steele, K. & Alexander, P. (1972) Thymus-derived lymphocytes produce an immunologically specific macrophage arming factor. *J. Exp. Med.*, **136**, 1318.

Fidler, I. J. (1975) Macrophage activation by lymphocyte supernatants. *J. Natl Cancer Inst.*, **55**, 1159.

FIDLER, I. J., DARNELL, J. H. & BUDMEN, M. B. (1976) *In vitro* activation of mouse macrophages by rat lymphocyte mediators. *J. Immunol.*, **117**, 666.

HERBERMAN, R. B. & HOLDEN, H. T. (1978) Natural cell-mediated immunity. *Adv. Cancer Res.*, **27**, 305.

JOASOO, A. & MCKENZIE, J. M. (1976) Stress and immune response in rats. *Int. Arch. Allergy Appl. Immunol.*, **50**, 659.

KELLER, R. (1973) Cytostatic elimination of syngeneic rat tumor cells *in vitro* by non-specifically activated macrophages. *J. Exp. Med.*, **138**, 625.

KELLER, R. (1978*a*) Macrophage-mediated natural cytotoxicity against various target cells *in vitro*. I. Macrophages from diverse anatomical sites and different strains of rats and mice. *Br. J. Cancer*, **37**, 732.

KELLER, R. (1978*b*) Macrophage-mediated natural cytotoxicity against various target cells *in vitro*. II. Macrophages from rats of different ages. *Br. J. Cancer*, **37**, 742.

KOOPMAN, J. P. & JANSSEN, F. G. J. (1974) The suitability of an intestinal flora with colonization resistance factor for SPF mice, rats and gerbils. *Z. Versuchstierk*, **16**, 164.

KRAHENBUHL, J. L. & LAMBERT, L. H. (1975) Cytokinetic studies on the effects of activated macrophages on tumor target cells. *J. Natl Cancer Inst.*, **54**, 1433.

KRAHENBUHL, J. L., LAMBERT, L. H. & REMINGTON, J. S. (1976) The effects of activated macrophages on tumor target cells: escape from cytostasis. *Cell. Immunol.*, **25**, 279.

KRAHENBUHL, J. L. & REMINGTON, J. S. (1974) The role of activated macrophages in specific and nonspecific cytostasis of tumor cells. *J. Natl Cancer Inst.*, **113**, 507.

LEJEUNE, F. J. & REGNIER, R. (1975) Foetal bovine serum (FBS) induced cytostatic effect of peritoneal macrophages against mouse melanoma. *Behring Inst. Mitt.*, **56**, 28.

LEJEUNE, F. J., BEAUMONT, L., GARCIA, Y. & REGNIER, R. (1978) Peritoneal macrophage cytotoxicity induced by serum *in vitro*. *Biol. Med.*, **28**, 48.

LEONARD, E. J. & SKEEL, A. H. (1978) Isolation of macrophage stimulation protein (MSP) from human serum. *Exp. Cell Res.*, **114**, 117.

LI, J., MANSFIELD, J. M. & WALLACE, J. H. (1977) Tumorstatic effects of non-immune BALB/c peritoneal macrophages on syngeneic lymphoma cells *in vitro*. *Oncology*, **34**, 245.

MELSOM, H. & SELJELID, R. (1973) The cytotoxic effect of mouse macrophages on syngeneic and allogeneic erythrocytes. *J. Exp. Med.*, **137**, 807.

MELTZER, M. S. (1976) Tumoricidal responses *in vitro* of peritoneal macrophages from conventionally housed and germ-free nude mice. *Cell. Immunol.*, **22**, 176.

PEARSALL, N. N. & WEISER, R. S. (1970) *The Macrophage*. Philadelphia: Lea and Febiger. p. 7.

PELS, E. & DEN OTTER, W. (1974) The role of a cytophilic factor from challenged immune peritoneal lymphocytes in specific macrophage cytotoxicity. *Cancer Res.*, **34**, 3089.

PELS, E. & DEN OTTER, W. (1976) Demonstration of specific macrophage arming factor and spontaneous macrophage cytotoxicity. *IRCS Med. Sci.*, **4**, 385.

RASMUSSEN, JR., A. F. (1969). Emotions and immunity. *Ann. New York Acad. Sci.*, **164**, 458.

RYGAARD, J. & POVLSEN, C. O. (1974) (Eds.) *Proc. 1st Int. Workshop Nude Mice*. Stuttgart: Gustav Fisher Verlag.

TOBACH, E. & BLOCH, H. (1956) Effect of stress by crowding prior to and following tuberculous infection. *Am. J. Physiol.*, **187**, 399.

VAN DER WAAY, D., BERGHUIS-DE VRIES, J. M. & LEKKERKERK-VAN DER WEES, J. E. C. (1971) Colonization resistance of the digestive tract in conventional and antibiotic-treated mice. *J. Hyg.* (*Camb.*), **69**, 405.

WENSINCK, F. & RUSELER-VAN EMBDEN, J. G. H. (1971) The intestinal flora of colonization-resistant mice. *J. Hyg.* (*Camb.*), **69**, 413.

ZIEGLER, F. G., LOHMANN-MATTHES, M. L. & FISCHER, H. (1975) Studies on the mechanism of macrophage mediated cytotoxicity. *Int. Archs. Allergy Appl. Immunol.*, **48**, 182.